# The Attributable Direct Medical Cost of Healthcare Associated Infection Caused by Multidrug Resistance Organisms in 68 Hospitals of China

**DOI:** 10.1155/2019/7634528

**Published:** 2019-03-05

**Authors:** Huixue Jia, Weiguang Li, Tieying Hou, Hongqiu Ma, Yun Yang, Anhua Wu, Yunxi Liu, Jianguo Wen, Huai Yang, Xiaoli Luo, Yawei Xing, Weihong Zhang, Yinghong Wu, Lili Ding, Weiping Liu, Ling Lin, Ying Li, Meilian Chen, Liuyi Li

**Affiliations:** ^1^Peking University First Hospital, Beijing 100034, China; ^2^Shandong Provincial Hospital, Jinan 250021, China; ^3^Guangdong General Hospital, Guangzhou 510008, China; ^4^The First Affiliated Hospital of Anhui Medical University, Hefei 230022, China; ^5^The First Hospital, Shanxi Medical University, Taiyuan 030001, China; ^6^Xiangya Hospital, Central South University, Changsha 410008, China; ^7^General Hospital of PLA, Beijing 100853, China; ^8^The First Affiliated Hospital of Zhengzhou University, Zhengzhou 450052, China; ^9^Guizhou Provincial People's Hospital, Guiyang 550002, China; ^10^Jiangxi Province Children's Hospital, Nanchang 330006, China; ^11^Fourth Hospital of Hebei Medical University, Shijiazhuang 050019, China; ^12^Jiangsu Province Hospital, Nanjing 210029, China; ^13^Peking University People's Hospital, Beijing 100044, China; ^14^The First Affiliated Hospital of Xinjiang Medical University, Urumqi 830054, China; ^15^Inner Mongolia People's Hospital, Hohhot 010017, China; ^16^Heilongjiang Province Center for Disease Control and Prevention, Harbin 150030, China

## Abstract

Healthcare associated infection (HAI) is known to increase the economic burden of patients while the medical cost due to MDRO HAI is even higher. Three hundred eighty-one multidrug resistance organisms (MDROs) healthcare associated infection (HAI) case-patients and three hundred eighty-one matched control-patients were identified between January and December in 2015. The average total hospitalization medical cost of the case group was $6127.65 and that of the control group was $2274.02. The difference between the case group and the control group was statistically significant (t = 21.07; P < 0.01). The attributable cost of MDRO HAI was $3853.63. The direct medical costs due to different MDRO infections were different. The increased medical costs of CR-AB, CR-KP, and CR-PA were significantly higher than that of MRSA, MRSE, ESBL E. coli, and ESBL Kp (P < 0. 05). Among the subitem expenses, the drug cost increased the most (the average cost was $1457.72), followed by the treatment fee and test fee; the differences were statistically significant (P < 0.01).

## 1. Introduction

The cost of healthcare associated infection (HAI) has been reported in the United States since the 1970s, yet there were few studies on the economic evaluation of HAI in other countries [1–6].

At present, there are many studies on the economic loss of HAI. The subjects and methods are different [7–11]. After matching the factors of influencing medical cost and hospitalization time, a comparison was performed between HAI and uninfected patients. Most of the studies used retrospective epidemiological methods and occasionally had both prospective research and decision-making models. Because of the difference of research methods, the estimated results of economic loss caused by HAI fluctuate greatly [12, 13]. The factors influencing the results includes sample size, hospital characteristics, total number of patients, source of infection, economic model, payment method, diagnostic criteria for HAI, etc. Robert W. Haley, scientist for Emory University School of Medicine, found that studies comparing the cost of matching HAIs with uninfected cases may exaggerate the true cost of HAI [14]. However, this method is still the most widely used method.

The economic loss caused by HAI includes direct economic loss and indirect economic loss. For patients, direct economic loss includes direct medical cost loss and direct nonmedical cost loss. Direct medical costs include bed charges, testing fees (such as blood, biochemistry, microbiology, radiology, etc.), antimicrobial and other drugs, treatment, surgery, blood transfusions, oxygen transfusions, the increase in nutritional support costs, and the increase in medical costs for basic diseases due to HAI. The use of antimicrobial drugs is the main reason for the increase of medical cost of HAI followed by the increase of treatment cost due to the prolongation of hospital stay. Direct nonmedical expense losses are reflected in increased care expenses, board expenses, etc. Indirect economic loss mainly refers to the prolonged hospital stay due to HAI, the delayed work expenses of patients and family members, and the decrease of income due to the decline or even loss of labor ability in the later stage of the patients. Research on direct nonmedical costs and indirect economic losses is relatively rare.

At present, there are many research studies on the economic burden caused by infection in different sites [15–22]. With the application of broad-spectrum antimicrobial agents, the rate of bacterial resistance is increasing [23]. Some research [24, 25] indicated that Multidrug-Resistant Organism (MDRO) infection can increase the direct economic burden of hospitals and patients.

In a report to the Canadian Committee on Antibiotic Resistance, it provided a comprehensive assessment of the human and financial burden from drug-resistant infections in Canada [26] with a focus on hospital-associated infections, especially methicillin-resistant Staphylococcus aureus. Results indicated that antimicrobial resistance adds $8.7 to $13.9 million more in direct costs than if these infections were drug susceptible, plus costs of screening programs (another $10.3 million) and measures to contain carriers detected ($15.9 million more). If American prevalence is applied, direct cost rises to $103.9 to $187.1 million (approximately $63.9 to $102.2 million more than for similar susceptible infections), screening cost remains $10.3 million, and cost to contain colonized carriers rises to $157.2 million.

Most of the published literatures were conducted in one center or involved only single multidrug-resistant bacteria, or even a multidrug-resistant bacteria infection from a single site. There is still a lack of medical cost research of healthcare associated infection (HAI) caused by different MDROs in a large sample; therefore the results are quite different. It was not possible to compare the differences in medical costs due to different multidrug-resistant organisms healthcare associated infections.

## 2. Materials and Methods

### 2.1. Sampling Methods

**Figure 1:**
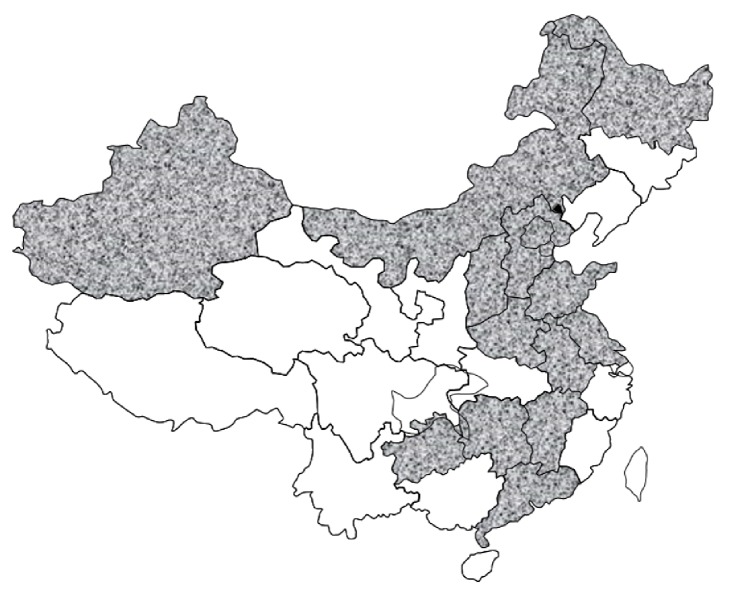
Geographic locations of China and 14 primary sampling provinces (dashed areas).

This survey was conducted in 68 hospitals in 14 primary sampling provinces (PSPs; Figure 1) of 7 major regions of China (Northeast, North, Central, East, South, Northwest, and Southwest). Each province had at least one provincial or ministerial level general hospital, one prefectural or municipal level general hospital, and/or one district or county level general hospital.

### 2.2. Patients

Three hundred eighty-one multidrug-resistant organisms (MDROs) healthcare associated infection (HAI) case-patients and three hundred eighty-one matched control-patients were identified between January and December in 2015. The matching principle included the same sex, the age difference less than 5 years, the same or similar first diagnosis (the main diseases in the hospital), and the same or similar surgical procedure that the case-patients had. The control-patients should have been hospitalized for more than 48 hours so that no healthcare associated infections occur.

### 2.3. MDROs Species

This study mainly monitored methicillin-resistant Staphylococcus aureus (MRSA), methicillin-resistant Staphylococcus epidermidis (MRSE), vancomycin resistant Enterococcus (VRE), extended-spectrum *β*-lactamases producing (ESBLs) Escherichia coli and Klebsiella pneumoniae, carbapenem-resistant Escherichia coli (CR-E. coli) and Klebsiella pneumoniae (CR-Kp), carbapenem-resistant Acinetobacter baumannii (CR-AB), and carbapenem-resistant Pseudomonas aeruginosa (CR-PA).

### 2.4. Definition

Healthcare associated infections were defined using the nosocomial infection diagnostic standard (trial) issued by the Ministry of Health of China in 2001 [27].

### 2.5. Measurement of MDROs HAIs Associated Direct Medical Cost

Check the medical records of case-patients and control-patients and investigate the medical expenses including bed, nursing, medication, antimicrobial, operating, therapeutic, laboratory, and inspection costs, based on the November 2018 exchange rate of 6.92 RMB to the US dollar, converting the cost into dollars.

### 2.6. Data Analysis

The patients with MDRO HAIs were assumed as the case group and patients without any HAIs as the control group. The total medical cost was evaluated for the case. The descriptive statistics and frequency distribution such as mean, standard deviation, and percentage were used.

The differences were analyzed by matched T tests. Due to the skewed distribution of the medical expenses of the patients, the matched T test was made after logarithmic conversion. All statistical analyses were two sided, and P < 0.05 was considered significant. Also, SPSS, version 18, was used for data analysis.

## 3. Results

A total of 68 hospitals in 14 provinces were investigated, including 16 (23.53%) provincial or ministerial hospitals, 28 (41.18%) prefectural or municipal hospitals, and 24 (35.29%) district or county level hospitals.

Of 381 case-patients, 60 (15.75%) cases detected MRSA, 18 (4.72%) cases detected MRSE, 124 (32.55%) cases detected ESBL E. coli, 38 (9.97%) cases detected ESBL Kp, 11 (2.89%) cases detected VRE, and 49 (12.86%) cases detected CR-AB. 28 (7.35%) cases detected CR-E. coli, 25 (6.56%) cases detected CR-Kp, and 28 (7.35%) cases detected CR-PA.

The average total hospitalization medical cost of the case group was $6127.65 and the control group was $2274.02. The difference between the case and control group was statistically significant (t = 21.07. P < 0.01). The attributable medical cost of MDRO HAI was $3853.63.

The medical cost due to different MDRO infection was different. The increase of medical cost in VRE ($8741.60) infection was the greatest, followed by CR-AB infection and CR-Kp infection; the increased medical cost was $8527.28 and $7947.24, respectively. The increased medical cost of ESBL Kp infection ($2136.81) was the least. The medical cost in all MDRO infection groups was significantly higher than that of the control group (P < 0. 01). Compared between different MDRO HAIs, the increased medical costs of CR-AB, CR-KP, and CR-PA were significantly higher than that of MRSA, MRSE, ESBL E. coli, and ESBL Kp (P < 0. 05). The increased medical costs of VRE (t=1.92; P=0.06) and CR-E. coli (t=1.87; P=0.07) were higher than that of ESBL Kp. There was no significant difference among other MDROs (Table 1).

The additional hospital expenses due to MDRO HAI increased $2148.76 in district or county hospitals, $4753.30 in prefectural or municipal hospitals, and $5401.82 in provincial or ministerial hospitals. The medical cost in the case group was significantly higher than that of the control group (P < 0. 01). There was no significant difference in the medical cost between different hospital levels (P > 0. 05) (Table 2).

The additional direct medical cost due to MDRO HAI in different regions was $2780.50 to $6029.58. East China is the least; Northwest China is the greatest. The medical cost in the case group was significantly higher than that of the control group (P < 0. 01) (Table 3).

Among the subitem expenses, the medication cost increased the most; the average was $1457.72. The average cost of antimicrobial drugs increased $367.48 followed by the treatment fee and test fee. The differences were statistically significant (all P < 0.01) (Table 4).

## 4. Discussion

Healthcare associated infection (HAI) is known to increase the economic burden of patients while the cost of MDRO HAI is higher. More than 70% of the bacteria that cause HAIs are resistant to at least one of the drugs most commonly used to treat them [28, 29] and there is strong evidence that the attributable costs, length of stay, and mortality are even greater when an infection is caused by a MDRO [30–36]. For many patients, inadequate or delayed therapy and severe underlying disease are primarily responsible for the adverse outcomes of infections caused by a MDRO. Patients with infections due to antimicrobial-resistant organisms have higher costs (~$6,000–$30,000) than do patients with infections due to antimicrobial-susceptible organisms; the difference in cost is even greater when patients infected with antimicrobial-resistant organisms are compared with patients without infection [32].

The main purpose of this study was to investigate the increase of hospitalization medical costs of nine MDRO-induced HAIs compared with noninfectious patients. The average increased cost was USD $3853.63. Although the increased rate of district or county hospitals was lower than that of provincial and municipal hospitals, there was no statistical difference. It shows that HAI caused by MDRO can cause similar increases in hospitalization medical costs in any level of hospital.

The direct medical costs due to different MDRO infection increases were different, ranging from $2136.81 to $8741.60, with an average of $3853.63, which was $1853.75 higher than that caused by overall HAI [15]. It shows that the occurrence of MDRO HAIs will bring more additional medical cost to patients, which suggests that we should strengthen the prevention and control of MDRO HAI.

The increased medical cost of VRE infection was the highest. It was $8741.60, which was lower than that of Xiaoyan Song's study ($77558) [33]. The additional medical costs of CR-AB infection and CR-Kp infection were $8527.28 and about $7947.24, respectively, which was far lower than that for MDR-AB in Stephen J. Wilson's study ($98575) [34] which is even lower than a Chinese research study which was conducted in a clinical center [37]. In addition, ESBL Kp infection incurred the least medical cost which was $2136.81. One MDRO that has received much attention is methicillin-resistant Staphylococcus aureus (MRSA). The increased disease burden of MRSA compared with infections caused by methicillin-susceptible S. aureus has been confirmed in two meta-analyses [38, 39]. The increased attributable cost of MRSA versus methicillin-susceptible S. aureus was estimated to be $4000~ $40000 per infection [31, 40]. In this study the increased medical cost caused by MRSA HAI was $3631.94. The difference with other international research studies may be related to the charging mechanism and living standard of different countries.

It can also be seen from the study that the increase of direct medical costs caused by carbapenem-resistant MDROs, such as CR-AB, CR-Kp, and CR-PA, was significantly higher than that of other MDROs, suggesting that carbapenem-resistant MDROs are more difficult to treat clinically and the cost of treatment is higher. It is suggested that, in the course of daily diagnosis and treatment, this kind of bacteria should be regarded as the most important part of prevention and control and the use of carbapenem should be more carefully considered during the application of antimicrobial agents.

In addition, by analyzing the composition of the increased medical expenses, it is found that the cost of medicine, treatment, and laboratory examination increased the most which is consistent with the results of most studies in China. In order to make a definite diagnosis after infection more tests are needed with the most important one being laboratory examination. The use of antimicrobial agents for the treatment of infection also increases the cost of medicine and treatment. Foreign studies [41] have found that the cost of antibiotics, nursing, disinfection, and sterilization and the consumption of disposable medical and sanitary supplies are the main factors that cause the additional expenses of healthcare associated infection. This difference is due to many of the costs of disinfection and sterilization and disposable medical and sanitary supplies in China cannot be charged to patients and are expenses incurred by medical institutions. If the costs of this part are increased, healthcare associated infections can cause more direct medical costs to patients. A report [26] confirmed prior research showing psychological harm from both infection control practices and serious underlying disease resulting in depression and fears that extend beyond discharge from the hospital. Infection control measures and the impact of resistance on the ability of acute care hospitals to transfer patients ready for discharge constitute a larger part of direct cost than antibiotic treatment itself.

There are also some deficiencies in this study: there is no comparison of the increase of medical cost caused by drug-resistant bacteria and sensitive bacteria. Therefore the analysis of the effect of drug-resistant bacteria on patients' economic loss is deficient and insufficient. However, the influence of drug-resistant bacteria on the medical cost and the differences of different drug-resistant bacteria are also obtained from a certain level which also indicates that we should strengthen the prevention and control of multidrug-resistant bacteria and the rational use of antimicrobial agents, especially the prevention and control of carbapenem-resistant pathogens and the management of carbapenem antibiotics.

## 5. Conclusions

MDRO HAI can significantly increase the direct medical cost of patients which is higher than that caused by overall healthcare associated infection. There were differences in the increased direct medical costs of different multidrug-resistant bacteria infection with a more significant cost increase in the carbapenem-resistant MDROs. Through this study, it is suggested that we should focus our efforts towards prevention and control of MDRO HAI, especially carbapenem-resistant MDROs to reduce cost and improve outcomes. In addition, controlling of cost can be a challenging in today's market; however cost reduction can be achieved through more of a preventative approach in both antimicrobial stewardship and preventing transmission of MDRO in healthcare.

## Figures and Tables

**Table 1:** Direct medical cost due to different MDRO's.

MDRO	n	Case group (Average, US$)	Control group (Average, US$)	Attributable medical cost (Average, US$)	t	*P*
VRE	11	12580.38	3838.79	8741.60	5.75	<0.01
MRSA	60	6287.73	2655.79	3631.94	7.35	<0.01
MRSE	18	4546.67	1934.61	2612.06	5.53	<0.01
CR-AB	49	11526.41	2999.12	8527.28	9.17	<0.01
CR-Kp	25	10646.18	2698.94	7947.24	8.78	<0.01
CR-PA	28	9512.48	1968.31	7544.17	7.81	<0.01
CR-E. coli	28	6280.50	2061.55	4218.95	5.78	<0.01
ESBL E. coli	124	4216.91	1959.72	2257.19	10.64	<0.01
ESBL Kp	38	4204.30	2067.49	2136.81	5.94	<0.01

**Table 2:** Direct medical cost due to MDRO HAI in different hospital levels.

Level	N	Case group (Average, US$)	Control group (Average, US$)	Attributable medical cost (Average, US$)	t	P
Provincial or ministerial	130	8419.65	3017.82	5401.82	12.58	<0.01
Prefectural or municipal	129	7467.82	2714.53	4753.30	13.02	<0.01
District or county	122	3543.18	1394.42	2148.76	10.88	<0.01

**Table 3:** Direct medical cost due to MDRO HAI in different regions.

Regions	N	Case group (Average, US$)	Control group (Average, US$)	Attributable medical cost (Average, US$)	t	P
Northwest	53	8523.33	2493.75	6029.58	8.22	<0.01
South	32	8146.20	2260.72	5885.47	7.99	<0.01
Central	40	7059.94	2651.37	4408.58	8.25	<0.01
North	65	7000.73	2837.68	4163.05	8.26	<0.01
Northeast	14	6809.48	2758.01	4051.48	3.40	<0.01
Southwest	26	5767.40	2235.92	3531.48	7.72	<0.01
East	151	4675.55	1895.04	2780.50	11.86	<0.01

**Table 4:** The subitem of direct medical cost due to MDRO HAI.

Subitem	N	Case group (Average, US$)	Control group (Average, US$)	Attributable medical cost (Average, US$)	*t*	*P*
Medication cost	359	2208.99	751.27	1457.72	17.15	<0.01
Antimicrobial cost	75	556.28	188.80	367.48	6.42	<0.01
Therapeutic cost	336	481.05	142.85	338.20	14.10	<0.01
Laboratory cost	359	474.56	209.75	264.82	16.48	<0.01
Bed cost	336	165.61	58.79	106.82	19.42	<0.01
Operating cost	137	410.67	321.73	88.94	4.19	<0.01
Inspection cost	330	205.78	117.22	88.56	7.15	<0.01
Nursing cost	335	108.14	33.85	74.29	16.27	<0.01

## Data Availability

The data used to support the findings of this study are included within the article.
